# Review of Topical Treatment of Upper Tract Urothelial Carcinoma

**DOI:** 10.1155/2009/472831

**Published:** 2008-11-05

**Authors:** Kenneth G. Nepple, Fadi N. Joudi, Michael A. O'Donnell

**Affiliations:** Department of Urology, University of Iowa, 200 Hawkins Dr., 3 RCP, Iowa City, IA 52242-1089, USA

## Abstract

A select group of patients with upper tract urothelial carcinoma may be appropriate candidates for minimally invasive management. Organ-preserving endoscopic procedures may be appropriate for patients with an inability to tolerate major surgery, solitary kidney, bilateral disease, poor renal function, small tumor burden, low-grade disease, or carcinoma in situ. We review the published literature on the use of topical treatment for upper tract urothelial carcinoma and provide our approach to treatment in the office setting.

## 1. Introduction

Nephroureterectomy with open excision of bladder cuff remains the standard of care for organ-confined upper tract urothelial cancer in patients with a normal contralateral kidney. Laparoscopic surgery has broadened the population of patients who are able to tolerate this surgery; however, due to concerns for preservation of renal function or inability to tolerate surgery, a selected group of patients may be suitable candidates for less-invasive management with endoscopic approach. Organ-preserving endoscopic procedures may be appropriate for patients with a solitary kidney, bilateral disease, poor renal function, or inability to tolerate major surgery [1, 2]. In addition, the indications for minimally invasive therapy have evolved to include small tumor burden, low-grade disease, or carcinoma in situ in patients with normal contralateral renal function [3]. Patients with high-grade disease, multifocal tumors, or history of recurrent tumor are not ideal candidates for topical therapy because of risk of recurrent or progressive disease. Patients managed with less invasive treatment must be made aware of the concern for progressive or recurrent disease. In addition, patients must be compliant with both the treatment regimen and the required subsequent follow-up.

The role of intravesical immunotherapy in non-muscle-invasive urothelial cancer of the bladder has been firmly established [4, 5]. A variety of agents have been utilized (Table 1), with BCG showing the greatest efficacy [4]. In the treatment of upper tract urothelial carcinoma, instillation of topical immunotherapy has been used as primary or adjuvant treatment, but upper tract treatment can be problematic because agents must be delivered to the renal pelvis and ureter to be effective. A challenge to the practicing urologist is finding a way to implement this treatment in the busy office setting. We review the published literature on topical treatment of upper tract urothelial cancer, and provide our approach to treatment in the office setting.

## 2. Upper Tract Evaluation

Patients must be properly selected for topical upper tract treatment. Upper tract abnormalities are often identified as a filling defect on delayed images of a CT urogram or IVP done for hematuria work-up or during selective upper tract washings for a positive bladder cytology in the absence of any bladder pathology. Our operative evaluation for positive cytology includes evaluation of both the bladder and upper tract for source of positive cytology. Initially, a rigid cystoscope is introduced and the bladder is inspected. The bladder is drained and a bladder wash with normal saline is obtained for cytology by rinsing the bladder several times. Cytology from the first kidney is obtained using normal saline washes through a 5 french open-ended ureteral catheter. To reduce the chance of bladder contamination, positive pressure saline is infused through the catheter up until the point in which it is introduced into the ureter The catheter is advanced up into the renal pelvis (~25 cm) and two 5–7 cc saline washes are obtained followed by additional washes at 20, 15, and 10 cm. The catheter is then readvanced up into the renal pelvis where the remaining fluid is aspirated and pooled together with the other washes. A retrograde pyelogram is then performed from the renal pelvis downwards as the catheter is removed. The same procedure is then performed on the contralateral upper tract. It should be noted that special care must be taken to avoid contamination of specimens by using different ureteral catheters for each side. If any abnormality is seen on retrograde pyelogram, then the upper tract should be evaluated with ureteroscopy with subsequent upper tract wash, brush biopsy, or tissue biopsy as deemed appropriate. The bladder should be inspected and five random bladder biopsies taken in a stellate manner (trigone, base, dome, and both lateral side walls). An essential component of a complete evaluation is to obtain a separately labeled biopsy from the prostatic urethra, which can serve as a sanctuary site for urothelial carcinoma.

Patients treated with topical infusion therapy typically have either carcinoma in situ or lesions that have been resected endoscopically. A mass in the collecting system or ureter identified on retrograde pyelogram or CT urogram prompts a focused operative evaluation. Small lesions may be amenable to endoscopic treatment (ureteroscopic or percutaneous) as discussed elsewhere in this special issue. It is highly unlikely that topical treatment by itself would eradicate radiographically visible disease. After primary treatment or the presence of hematuria, BCG treatment should be delayed 2 to 4 weeks to allow the urothelium to heal and decrease the likelihood of systemic side effects. Of note, in patients with positive cytology from both the bladder and upper tract with no mass lesion, the status of the upper tract should be considered inconclusive. In these cases, we generally start with intravesical treatment as the positive upper tract cytology could be due to contamination from the bladder. Restaging after intravesical therapy is prudent to reevaluate the upper tracts. If the bladder is disease-free and the upper tract cytology continues to be positive, then this rules out the possibility of contamination and should be treated accordingly.

## 3. Office-Based Approach for Upper Tract Treatment

**Figure 1:**
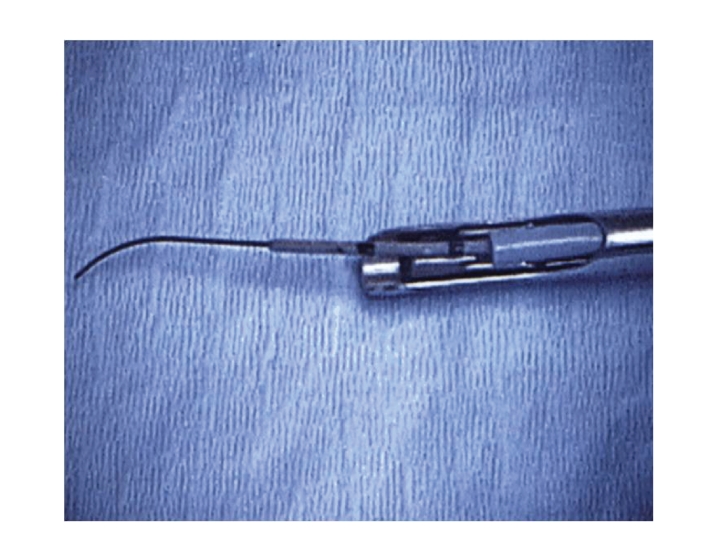


**Figure 2:**
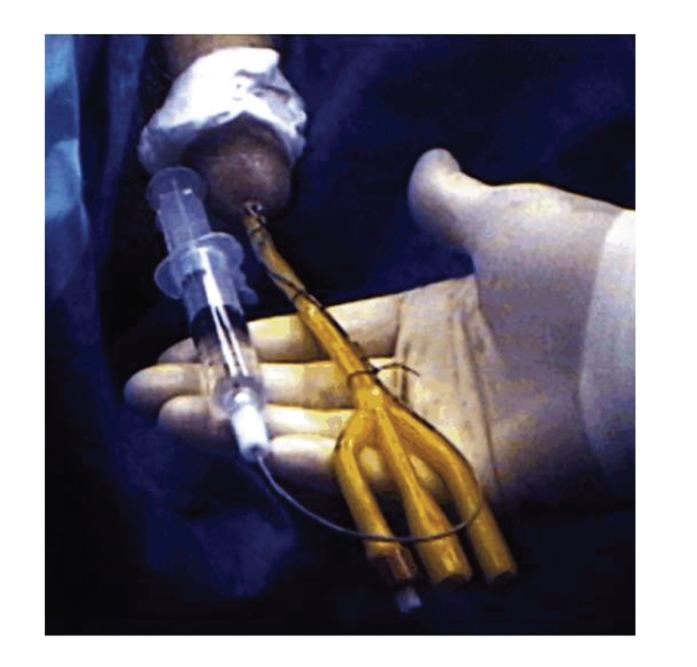


We use an office-based approach for placement of ureteral catheter(s) for upper tract therapy. Flexible cystoscopy is performed, sometimes with oral narcotic/benzodiazapine premedication or intravenous sedation (morphine, versed) based on patient preference. The ureteral orifice is visualized and cannulated with a 0.018 inch angled glidewire that is passed to the renal pelvis. A 4 F whistle-tip catheter is then passed over the glidewire (Figure 1). Under direct vision, the catheter is slid over the wire into the ureter and a second ureteral catheter with the tip cut off is used as a pusher to further advance the catheter. Using the catheter markings as a guide, the catheter is typically advanced to 25 cm to place it in the renal pelvis. The flexible scope is then carefully backed out leaving the catheter in the mid renal pelvis. The guidewire is subsequently removed once proper positioning is established. It is very helpful to use fluoroscopy, at least for the first session, to establish proper catheter position and rule out unexpected anatomical difficulties. Free flow of urine from the catheter or retrograde injection of contrast verifies proper position in the collecting system. Ureteral catheters are secured via silk ties to a foley catheter placed to drain the bladder and brought to rest at the bladder neck (Figure 2). The Foley catheter is either left to straight drainage or elevated over the bedrail to allow some collection into the bladder depending on whether simultaneous bladder exposure is desired. It may also be capped during treatment if formal intravesical instillation is performed at the same time. This particular technique of using a small caliber ureteral catheter over a small slippery wire is usually very atraumatic and allows free fluid flow around and out the splinted ureter. If trauma or bleeding is encountered, then treatment may need to be deferred in the case of BCG. In patients who have previously undergone cystectomy with urinary diversion, treatment is usually performed with percutaneous nephrostomy tube as retrograde access to the ureter is difficult.

We use a treatment regimen of low-dose BCG (one-third to one-tenth standard dose) plus interferon-alpha-2b (50–100 million units) in 50 cc normal saline based on the effectiveness of this combination in non-muscle-invasive bladder cancer [5]. The viscosity of the full-dose BCG suspension is such that it will not spontaneously drip under gravity instillation through such a small 4 french catheter. The patient is positioned supine. The medication is suspended in an IV bag no more than 30 cm above the kidney level. Medication is instilled via microdrip tubing at the rate of 1 drop per 2 seconds, corresponding to a rate of approximately 30 cc/h. Medication is only instilled via gravity and should never be placed on a pump due to concern for increased intrarenal pressure. At the conclusion of treatment, the foley catheter is drained and then removed, bringing the attached ureteral catheters out with it. If treatments are not able to be administered via ureteral catheters, then a percutaneous nephrostomy tube can be placed at the beginning of treatment and medicine can be instilled via nephrostomy tube, with the tube capped between weekly treatments.

Patients receive weekly treatment for 6 sessions over 6 weeks. Then, 6 weeks following the last treatment, patients are restaged with bilateral upper tract washings and retrograde pyelograms, bladder washing, and random bladder biopsies along with prostatic urethral biopsy. If the results are negative, consideration is given to 3 future maintenance treatments starting 6 weeks later. While there have been no published results on the efficacy of maintenance treatment, the addition of 3 weekly maintenance treatments is well established for bladder CIS [6].

## 4. Literature Review

The true benefit of topical therapy, either as a primary treatment for carcinoma in situ or as an adjuvant for endoscopically treated tumors, is difficult to assess based on the variance in the reported literature. In contrast to bladder cancer, which is relatively common, upper tract urothelial cancer is uncommon and, therefore, a single center is not able to accrue significant numbers of patients for a prospective study. Additionally, heterogeneous groups of patients receive such therapy (solitary versus multifocal disease, primary versus recurrent, low- versus high-grade). Retrospective case reviews can also have methodological flaws. The goal is to maximize the effectiveness of treatment while minimizing side effects and complications. Multiple different treatment regimens have been utilized with BCG being used most commonly (Table 2). No randomized studies have been performed to evaluate such therapy, and most studies have set the number of instillations empirically based on expert opinion or extrapolating from intravesical treatment regimens.

## 5. BCG

In 1996, Yokogi et al. analyzed therapeutic outcomes of BCG perfusion therapy for upper urinary tract CIS in 8 renal units—5 through a percutaneous nephrostomy tube and 3 through a retrograde ureteral catheter [7]. Follow-up cystoscopy, retrograde pyelography, and selective urinary cytology were obtained 4 weeks after the last treatment and every 3 months thereafter. In 5 of 8 renal units, the cytology remained negative for 10 to 46 months after treatment, while the other 3 renal units had persistently positive cytology. Of 2 patients treated through a ureteral catheter, 1 developed a ureteral stricture and the other developed renal tuberculosis, which emphasizes that urologists must be mindful of the development of complications.

 In contrast, Nishino et al. used BCG perfusion treatment (instilled weekly for 4 or 8 weeks) to treat upper tract CIS via retrograde catheterization with either a 6 French ureteral catheter or an 8 French indwelling double J ureteral stent [8]. At a mean follow-up of 22 months (range 9–38 months), all 8 renal units had negative cytology, and cytology became negative after 1 or 2 instillations of BCG. However, 1 patient had recurrent CIS in the prostatic urethra treated with intravesical BCG instillation. Complications included ureteral stenosis in 2 patients and self-limited irritative symptoms occurred in all patients.

An indwelling ureteral stent was used to treat 11 patients with upper tract CIS as reported by Nonomura et al. in 2000 [9]. Reflux up the ureteral stent was confirmed using contrast at the time of initial ureteral stent placement. BCG was instilled into the bladder weekly, 6 times in total as 1 course. At the end of 1 course, 9 cases showed negative urinary cytology; however, 2 patients had recurrence in the upper urinary tract after 4 and 8 months, and repeat BCG therapy was not effective. Two patients never normalized their cytology. The mean recurrence-free time was 19.6 months. As side effects, 8 cases (72.7%) developed bladder symptoms, and 4 presented with fever higher than 38°C, but the authors reported that no patient needed antitubercular treatment.

The efficacy of retrograde flow to the upper tract via an indwelling double J ureteral stent has been questioned. Yossepowitch et al. used performed cystograms with an indwelling stent in place and reported that retrograde flow occurred in only 56% of patients. Additionally, the mean minimal intravesical volume to obtain reflux was 170 mL, which is higher than the typically instilled treatment volume [10].

Miyake et al. evaluated the efficacy of intrarenal BCG instillation for the treatment of CIS of the upper urinary tract [11]. Sixteen patients (17 renal units) were treated with BCG administered once weekly, 6 times in total using percutaneous nephrostomy tube in 5 patients, and a retrograde ureteral catheter in 11. During the median follow-up period of 30 months (range 9–90 months), 13 patients (14 renal units) remained cytologically negative. However, 1 of these 13 patients had CIS in the bladder and prostatic urethra 34 months after the BCG therapy and underwent radical cystectomy. Bladder irritability and fever higher than 38°C was observed in 12 and 9 patients, respectively; however, no patient received antitubercular treatment.

In the adjuvant setting after ureteroscopic tumor ablation, Patel and Fuchs in 1998 reported on the use of topical BCG therapy (indwelling stent and intravesical BCG in 3 renal units, and ureteral catheter instillation passed through a suprapubic stab incision in 14 which allowed the avoidance of weekly cystoscopy) [12]. At a mean follow-up of 15 months, 15 of 17 renal units were preserved and remained tumor-free. Patients in this series were followed with regular flexible ureteroscopy along with cytology washings in the clinic using topical anesthesia. The authors attributed the favorable outcome in preserving renal units to improved resections made possible by the development of small caliber ureteroscopes, improved optics, and new ablative energy sources such as the holmium:YAG laser.

In one of the largest series of topical upper tract treatment, Thalmann et al. (2002) retrospectively evaluated the results of BCG therapy for upper urinary tract disease in patients not eligible for nephroureterectomy [13]. Thirty-seven patients (22 with CIS, 15 with Ta or higher after endoscopic resection) were treated with 6 weekly perfusions of BCG via a 10 French nephrostomy tube. At a median follow-up of 42 months (range 8–137 months), 14 patients (38%) died of urothelial cancer, 11 (29%) of other causes, and 12 (33%) were alive. Other adverse outcomes included severe septicemia in 2 patients. There was no seeding of the nephrostomy tube tract and dialysis was avoided. Overall median survival was 42 months (range 1–137 months) with median recurrence-free survival of 21 months (range 1–137 months). The authors noted that this was a patient population with a poor prognosis and while BCG extended survival for some patients, it did not provide cure except for some patients with CIS. Of the patients treated in the adjuvant setting for papillary disease, only 13% remained without recurrent or progressive disease with a median time of recurrence of 10 months. In contrast, treatment of CIS resulted in 32% of renal units remaining disease-free for a median follow-up of 51 months.

## 6. BCG Plus Interferon

The data regarding supplemental interferon is not firmly established, validated, or widely used for bladder cancer and certainly not for upper tract transitional cell carcinoma. However, there have been two reports of its use in treating upper tract urothelial carcinoma. We have reported on 15 patients (23 renal units) with upper tract urothelial cancer after endoscopic resection who received 6 weekly adjuvant low-dose BCG (one-tenth standard dose) and interferon-*α* (100 million units) [14, 15]. Nineteen of the renal units had CIS, 2 had Ta grade 1, one had Ta grade 3, and one had T1 grade 3. Sixteen were treated with ureteral catheters while seven were treated with percutaneous nephrostomy tubes. At a mean follow-up of 15.3 months (range 3–44 months), the response rate was 70% (16 of 23 renal units). The highest response rate was in patients with CIS (14 of 19; 74%). One patient had BCG sepsis that required 6 months of antitubercular therapy.

In 2007, Katz et al. published their initial experience with upper tract BCG-IFN [16]. A series of 10 patients (11 renal units) received 6 weekly courses of BCG (half strength) plus IFN-*α*2b (50 million units) via ureteral catheter. With a median follow-up of 24 months, 80% demonstrated a complete response, while 20% had a partial response (decrease in tumor size, number, or both). The authors reported that the treatment was well tolerated in the office setting and did not note any complications.

## 7. Other Agents

Less information is available on the use of mitomycin C topical treatment. In 1997, Keeley and Bagley reported on adjuvant mitomycin C (40 mg in 3 divided doses via ureteral catheter) in 19 patients (21 renal units) for high volume, recurrent, or multifocal urothelial carcinoma [1]. No systemic side effects occurred during or after treatment with mitomycin C which was attributed to the high molecular weight of mitomycin and limited systemic absorption. Thirty-five percent had a complete response, 27% had a partial response (reduction in tumor size > 50%), and 38% had no response. Tumors with a complete response were of similar size and grade as those that did not respond as well. With a mean follow-up of 30 months, none of the patients suffered local disease progression or died of disease; however, nearly all of the patients required repeat ureteroscopic treatment for residual or recurrent disease.

Due to confounding variables and heterogeneous treatment groups, direct comparisons between treatment groups in a study can be difficult. One study in 1996 from Martínez-Piñeiro et al. reported on a series of upper tract carcinoma in which 26 patients received adjuvant supplemental topical therapy, and attempted to make comparisons between different treatments [17]. BCG and mitomycin C seemed to be most effective at preventing recurrences, with recurrence rates of 12.5% and 14.2%, respectively, compared to 60% for thiotepa. Fatal aplastic anemia from systemic absorption of MMC was reported in 1 patient. In 1996, Elliott et al. reported the Mayo Clinic experience with endoscopic treatment of upper tract urothelial carcinoma in which 18 of 44 patients received some form of topical therapy (BCG in 9, MMC in 5, thiotepa in 4) [18]. Their methods did not report mode of delivery. In their sample, a difference was not found in recurrence between those who did and did not receive adjuvant topical therapy. Similarly, Jarrett et al. reported that BCG therapy showed no significant improvement in survival in 19 of 30 renal units [19].

Similarly, comparisons between treatment and nontreatment groups may be complicated by selection bias. In 2004, Palou et al. presented results of the percutaneous approach to resection of upper urinary tract urothelial carcinoma in which 14 and 5 patients received BCG and MMC instillations, respectively [20]. Median time to recurrence was 24 months and the rate of kidney preservation was 74%. The authors reported a recurrence rate of 58% in those who received topical therapy compared to 27% in those who did not; however, the topical therapy group was higher risk with higher grade disease and comprised more patients with multiple tumors. There was a trend of recurrence in patients with multifocal tumors, history of bladder carcinoma in situ, and tumor in renal pelvis. The authors concluded that the percutaneous approach to renal urothelial tumor should be considered a valid option with a good long-term outcome; however, there was an obligation to a long-lasting, strict surveillance.

An alternative experimental treatment regimen in refractory patients is sequential gemcitabine and mitomycin C. Medication dosage is 1 gm gemcitabine in 50 cc phosphate-buffered normal saline, then 40 mg mitomycin C in 40 cc sterile water. The gemcitabine is instilled followed by the mitomycin immediately afterward. We reported in 2006 on a group of patients with treatment refractory non-muscle-invasive bladder cancer (8 of 37 had upper tract involvement [21]. Gemcitabine alone was effective in only 1 of 14 patients (7%) while sequential treatment with gemcitabine followed by mitomycin was successful in 13 of 23 patients (57%). The rationale for sequential treatment is that gemcitabine is too acidic and affects mitomycin activity if given together. Additionally, gemcitabine primarily kills cells undergoing DNA synthesis (S-phase) while mitomycin is non-cell phase-specific and leads to cell cycle arrest. Gemcitabine is given first followed by mitomycin to maximize therapeutic efficacy.

## 8. Conclusions

Without large prospective or randomized data, the topical treatment of upper tract urothelial cancer is to some extent anecdotal. Topical treatment of upper tract urothelial cancer has a role in selected patients, who must be committed to close follow-up because of the risk of recurrence and more concerning progression. The goal of treatment is to provide noninvasive, nephron-sparing treatment without compromising oncologic outcomes. Treatment can be successfully performed in the setting of a busy urologist's office. Further study is needed to identify the best candidates for this treatment approach and to determine which agents and schedule are most optimal. Our office-based approach to upper tract urothelial carcinoma provides the clinician with the framework to implement this treatment in practice.

## Figures and Tables

**Table 1:** Mechanism of immunotherapy agents.

BCG	Inflammatory host response
	Release of cytokines

Interferon	Lymphocyte activation
	Cytokine release
	Phagocyte stimulation
	Antiproliferative actions
	Antiangiogenic

Thiotepa	Alkylating agent
	Crosslinks nucleic acids

Mitomycin C	Inhibits DNA synthesis

Gemcitabine	Deoxyctidine analog
	Inhibits DNA synthesis

**Table 2:** Topical therapy of upper tract urothelial cancer.

Study	Indication	No. patients/no. renal units	Therapy	Mean follow-up, months	Comments
Jarret et al. 1995^19^	Adjuvant to percutaneous treatment	17 patients/19 renal units	BCG	55	No significant improvement in survival with BCG
Elliott et al. 1996^18^	Adjuvant to endoscopy	18 patients	BCG, thiotepa, MMC	NA	No difference in outcome between treated and untreated
Yokogi et al. 1996^7^	CIS	5 patients/8 renal units	BCG	10–46	NED in 5/8 renal units
Martinez-Pineiro et al. 1996^17^	Adjuvant to endoscopy	26 patients	BCG, MMC, thiotepa	31	12.5% recurrence with BCG, 14% with MMC, 60% with thiotepa
Keeley and Bagley, 1997^1^	Adjuvant to ureteroscopy	19 patients/21 renal units	MMC	30	35% complete response, 27% partial response, 38% no response
Patel and Fuchs, 1998^12^	Adjuvant to ureteroscopy	13 patients/17 renal units	BCG	15	NED in 15/17 renal units
Nishino et al. 2000^8^	CIS	6 patients/8 renal units	BCG	22	NED in 8/8 renal units
Nonomura et al. 2000^9^	CIS	11 patients	BCG	NA	NED in 7/11 patients
Burns et al. 2001^14^	CIS, adjuvant to endoscopy	15 patients/23 renal units	BCG-IFN	15	70% response rate
Thalmann et al. 2002^13^	Not eligible for open surgery	37 patients/41 renal units	BCG	42	87% recurred or progressed; 32% CIS were disease-free
Miyake et al. 2002^11^	CIS	15 patients/16 renal units	BCG	30	NED in 14/16 renal units
Palou et al. 2004^20^	Adjuvant to percutaneous treatment	19 patients	BCG in 14, MMC in 5	51	60% recurrence in treated patients vs 27% in untreated patients
Katz et al. 2007^16^	CIS, adjuvant to endoscopy	10 patients/11 renal units	BCG-IFN	24	80% complete response, 20% partial response

*BCG - bacillus Calmette-Guerin; MMC - mitomycin C; CIS - carcinoma in situ; IFN - interferon; NED - no evidence of disease; NA - not applicable.
